# Combination of Morphologic Criteria and α-Fetoprotein in Selection of Patients With Hepatocellular Carcinoma for Liver Transplantation Minimizes the Problem of Posttransplant Tumor Recurrence

**DOI:** 10.1007/s00268-014-2647-3

**Published:** 2014-05-24

**Authors:** Michał Grąt, Oskar Kornasiewicz, Zbigniew Lewandowski, Wacław Hołówko, Karolina Grąt, Konrad Kobryń, Waldemar Patkowski, Krzysztof Zieniewicz, Marek Krawczyk

**Affiliations:** 1 Department of General, Transplant and Liver Surgery, Medical University of Warsaw, 1A Banacha Street, 02-097 Warsaw, Poland; 2 Department of Epidemiology, Medical University of Warsaw, 3 Oczki Street, 02-007 Warsaw, Poland

## Abstract

**Background:**

Serum α-fetoprotein concentration (AFP) might be a useful addition to morphologic criteria for selecting patients with hepatocellular carcinoma (HCC) for liver transplantation (LT). The aim of this study was to evaluate the role of AFP in selecting HCC patients at minimal risk of posttransplant tumor recurrence in the setting of existing criteria.

**Methods:**

This retrospective cohort study was based on 121 HCC patients after LT performed at a single institution. AFP was evaluated as a predictor of posttransplant tumor recurrence with respect to fulfillment of the Milan, University of California, San Francisco (UCSF), and Up-to-7 criteria.

**Results:**

There was a nearly linear association between AFP and the risk of HCC recurrence (*p* < 0.001 for linear effect; *p* = 0.434 for nonlinear effect). AFP predicted HCC recurrence in patients (1) beyond the Milan criteria (*p* < 0.001; optimal cutoff 200 ng/ml); (2) within the UCSF criteria (*p* = 0.001; optimal cutoff 100 ng/ml) and beyond them (*p* = 0.015; optimal cutoff 200 ng/ml); and (3) within the Up-to-7 criteria (*p* = 0.001; optimal cutoff 100 ng/ml) and beyond them (*p* = 0.023; optimal cutoff 100 ng/ml) but not in patients within the Milan criteria (*p* = 0.834). Patients within either UCSF and Up-to-7 criteria with AFP level <100 ng/ml exhibited superior (100 %) 5-year recurrence-free survival—significantly higher than those within UCSF (*p* = 0.005) or Up-to-7 (*p* = 0.001) criteria with AFP levels higher than the estimated cutoffs or beyond with AFP levels less than the estimated cutoffs.

**Conclusions:**

Combining the UCSF and Up-to-7 criteria with an AFP level <100 ng/ml is associated with minimal risk of tumor recurrence. Hence, this combination might be useful for selecting HCC patients for LT.

## Introduction

Hepatocellular cancer (HCC) is the most frequent of the primary malignancies of the liver, ranked second and sixth on the list of the most common causes of tumor-related mortality worldwide in men and women, respectively [1]. Considering the unfavorable prognoses of untreated patients and only moderate response to chemotherapy [2, 3], surgical treatment is of the utmost importance. Unfortunately, given that the majority of these tumors occur in cirrhotic livers, the use of liver resection is often limited by the presence of portal hypertension and poor hepatic function, as reflected by the guidelines of the Barcelona Clinic Liver Cancer Group [4]. In contrast, liver transplantation (LT) not only removes all macroscopic and microscopic hepatic tumors with almost no risk of positive oncologic margins, it cures the underlying cirrhosis.

Early experiences with LT for HCC were characterized by an unacceptably high risk of posttransplant tumor recurrence and its related high mortality rate [5]. This situation was due to a lack of specific selection criteria and performing LT often in patients with large and multifocal tumors. A new era began with adoption of the Milan criteria into clinical practice (single tumor ≤5 cm or two or three tumors ≤3 cm, with no evidence of extrahepatic metastases or macrovascular invasion) [6]. Based on the results of several observational studies, expansions of these restrictive criteria have been proposed. One criteria set is that of the University of California, San Francisco (UCSF) with criteria of a single tumor <6.5 cm or two or three tumors <4.5 cm, total tumor diameter <8 cm. Another criteria set is the Up-to-7 criteria (size of the largest tumor + number of tumors ≤7) [7, 8]. Even though the conservative Milan criteria are still widely used for selecting patients with HCC to undergo LT, outcomes after LT for HCC remain inferior to those performed for benign indications [9, 10]. Because the inferior outcomes of HCC patients are mainly due to posttransplant tumor recurrence, the use of expansions of the Milan criteria that are already based solely on morphologic tumor characteristics would not eliminate these discrepancies. During this era of severe shortage of available organs and increasing demand for LT, there is general agreement in the transplant community that posttransplant outcomes of HCC patients should be similar to those undergoing transplantation for other indications [11]. Optimization of the existing selection criteria seems crucial to achieve this goal.

Historically, the serum α-fetoprotein (AFP) concentration was applied for HCC screening among high-risk groups and to establish the diagnosis. Considering its poor diagnostic features, it is no longer recommended in these clinical settings [12, 13]. Nevertheless, AFP is currently gaining an increasing role as a marker of biologic aggressiveness of tumors. Numerous studies confirmed the prognostic significance of preoperative AFP in both liver resection and LT [14–18]. However, there is no agreement as to the most appropriate cutoff for AFP when selecting HCC patients for LT [11]. The purpose of this study was to evaluate the exact role of AFP in selecting HCC patients at minimal risk of posttransplant tumor recurrence in the setting of the existing criteria.

## Material and methods

A total of 1,115 LTs were performed in the Department of General, Transplant, and Liver Surgery at the Medical University of Warsaw between December 1994 and June 2012. Basing on the results of explant pathology, 121 HCC patients were included in this retrospective cohort study. Patients with combined hepatocellular/cholangiocellular cancer (*n* = 3), fibrolamellar HCC (*n* = 3), sarcomatous HCC (*n* = 1), or macroscopic vascular invasion (*n* = 6) were excluded. The ethics committee of the Medical University of Warsaw approved the study protocol. Details on the operative technique, postoperative immunosuppression, and follow-up visits were described previously [15, 19].

The last available pretransplant AFP was collected (usually measured within 24 h preceding transplantation). Tumor recurrences at 3 and 5 years were set as primary and secondary end points of the study, respectively. They were used to calculate the corresponding recurrence-free survival rates (observations were censored at the time of the last follow-up or death caused by conditions other than tumor recurrence). In general, risk factors for HCC recurrence at 3 years were established in univariate analyses. Subsequently, the associations between AFP and hazard of recurrence were adjusted for the impact of potential confounders in a series of bivariate analyses. Establishment of the optimal cutoffs for AFP for predicting HCC recurrence at 3 years was based on true-positive (TP) and false-positive (FP) prediction rates derived from estimated 3-year recurrence-free survival rates of patients with AFP levels higher and lower than the following values: 10, 20, 35, 50, and 100–1000 ng/ml (at 100 ng/ml intervals). TP and FP rates were calculated using the following equations:

$$\begin{aligned} {\text{TP }} & = \, {{\left[ {a \times \, \left( { 1 { }{-}{\text{ RFSa}}} \right)} \right]} \mathord{\left/ {\vphantom {{\left[ {a \times \, \left( { 1 { }{-}{\text{ RFSa}}} \right)} \right]} {\left[ {\left( {a \times \, \left( { 1 { }{-}{\text{ RFSa}}} \right)} \right) {+} \left( {u \times \, \left( { 1 { }{-}{\text{ RFSu}}} \right)} \right)} \right]}}} \right. \kern-0pt} {\left[ {\left( {a \times \, \left( { 1 { }{-}{\text{ RFSa}}} \right)} \right) {+} \left( {u \times \, \left( { 1 { }{-}{\text{ RFSu}}} \right)} \right)} \right]}} \\ {\text{FP }} & = {{\left( {a \times {\text{ RFSa}}} \right)} \mathord{\left/ {\vphantom {{\left( {a \times {\text{ RFSa}}} \right)} {\left( {a \times {\text{ RFSa }} + u \times {\text{ RFSu}}} \right)}}} \right. \kern-0pt} {\left( {a \times {\text{ RFSa }} + u \times {\text{ RFSu}}} \right)}} \, \\ \end{aligned}$$

where *a* is the number of patients with AFP above the analyzed value at risk at the start of the observation period; *u* is the number of patients with AFP under the analyzed value at risk at the start of the observation period; RFSa is the estimated recurrence-free survival rate at 3 years for patients with AFP above the analyzed value; RFSu is the estimated recurrence-free survival rate at 3 years for patients with AFP under the analyzed value.

To the best of our knowledge, this novel methodologic approach allowed estimation of the optimal AFP cutoff values for predicting the 3-year recurrence rate even though the observation period was less than 3 years for some patients. For this reason, receiver operating characteristics curves were not used. Notably, the evaluation of the risk factors for HCC recurrence and AFP cutoffs was based on the first three posttransplant years when the vast majority of recurrent tumors are diagnosed [20]. Nevertheless, recurrence-free survivals of patients whose AFP levels were below and above the estimated AFP cutoffs were validated also within a time frame of five posttransplant years.

Data are presented as the median (range) for continuous variables and the number (%) for categoric variables. Associations between AFP and categoric and continuous variables were assessed with the Mann–Whitney *U* test and Spearman’s correlation coefficient, respectively. Survival estimates were based on the Kaplan–Meier method. Survival curves were compared using a log-rank test. Cox’s proportional hazards regression was used to evaluate risk factors for HCC recurrence in univariate and bivariate analyses. Linearity of the associations between AFP and recurrence hazard was assessed in general additive models and presented as spline functions. Hazard ratios (HRs) were presented with 95 % confidence intervals (95 % CI). The level of statistical significance was set at 0.05. All analyses were computed in SAS version 9.3 (SAS Institute, Cary, NC, USA) and S-Plus version 6.1 (TIBCO Software, Palo Alto, CA, USA).

## Results

Baseline characteristics of the 121 patients included in the study cohort are shown in Table 1. Median follow-up for the entire study cohort was 30 months. There was a positive correlation between AFP and the size of the largest tumor that verged on significance (*p* = 0.050) (Table 2). Significantly higher AFP was also noted in patients of female sex (*p* = 0.009), with hepatitis C virus-related cirrhosis (*p* = 0.010), without hepatitis B virus infection (*p* = 0.032), or with nonincidental tumors (*p* = 0.024) (Table 3). Although the associations between AFP and microvascular invasion (*p* = 0.252) or tumor differentiation (*p* = 0.775) did not reach the level of significance, the median AFP level was markedly higher for patients with microvascular invasion or poorly differentiated tumors.

**Table 1:** Characteristics of 121 patients included in the study cohort

Factor	Median (range) or no. (%)
General factors	
Recipient age (years)	55 (20–67)
Recipient sex	
Female	29 (24.0 %)
Male	92 (76.0 %)
Child-Turcotte-Pugh class	
A	54 (44.6 %)
B	50 (41.3 %)
C	17 (14.1 %)
Model for end-stage liver disease (points)	11 (6–40)
Hepatitis C virus infection	77 (63.6 %)
Hepatitis B virus infection	45 (37.2 %)
Alcoholic cirrhosis	26 (21.5 %)
Donor age (years)	48 (16–70)
Donor sex	
Female	33 (27.3 %)
Male	88 (72.7 %)
Cold ischemia (hours)	7.9 (2.9–11.8)
Fulfillment of selection criteria	
Milan	67 (55.4 %)
UCSF	83 (68.6 %)
Up-to-7	90 (74.4 %)
Tumor-related factors	
Serum AFP concentration (ng/ml)	23.7 (1.4–36208.0)
Size of largest tumor (cm)^a^	3.4 (0.3–14.0)
No. of tumors^a^	2 (1–10)
Total tumor volume (cm^3^)^a,b^	22.5 (0.02–5277.9)
Microvascular invasion^a^	37 (31.6 %)
Poor tumor differentiation^a^	13 (10.7 %)
Bilateral tumors^a^	33 (27.3 %)
Neodjuvant therapy	42 (34.7 %)
Chemoembolization	22 (18.2 %)
Ablation	13 (10.7 %)
Both	7 (5.8 %)
Salvage liver transplantation	9 (7.4 %)
Incidental tumors	13 (10.7 %)
Intraoperative factors	
Surgical technique	
Piggyback	101 (83.5 %)
Conventional	20 (16.5 %)
Packed RBC transfusions (units)	4 (0–48)
FFP transfusions (units)	8 (0–50)
Total operative time (hours)	7.0 (3.8–12.0)
Duration of hepatectomy (hours)	3.0 (1.5–5.1)
Warm ischemia (minutes)	68.5 (25.0–195.0)

*UCSF* University of California, San Francisco, *AFP* α-fetoprotein, *RBC* red blood cell, *FFP* fresh frozen plasma

^a^Based on explant pathology

^b^Calculated as a sum of estimated volumes of each tumor (4/3πr^3^)

**Table 2:** Associations between serum AFP concentration and other continuous variables

Variable 1	Variable 2	Correlation coefficient	*p*
AFP	Recipient age	0.046	0.627
AFP	Model for end-stage liver disease score	–0.037	0.691
AFP	Size of the largest tumor	0.185	0.050
AFP	No. of tumors	0.022	0.816
AFP	Total tumor volume	0.159	0.100

**Table 3:** Associations between serum AFP concentration and categoric variables

Variable	AFP (ng/ml)		
	Median	Range	*p*
Recipient sex			0.009
Female	124.3	3.3–17500.0	
Male	15.5	1.4–36208.0	
Child-Turcotte-Pugh class			0.173
A	30.7	1.6–36208.0	
B	34.5	2.0–7053.0	
C	9.2	1.4–17500.0	
Hepatitis C virus infection			0.010
No	10.6	1.9–7053.0	
Yes	43.1	1.4–36208.0	
Hepatitis B virus infection			0.032
No	41.8	1.4–36208.0	
Yes	10.2	1.9–23231.0	
Alcoholic cirrhosis			0.358
No	28.2	1.6–36208.0	
Yes	16.0	1.4–7053.0	
Milan criteria			0.196
Within	18.8	1.6–813.3	
Beyond	39.5	1.4–36208.0	
UCSF criteria			0.648
Within	25.2	1.6–23231.0	
Beyond	21.4	1.4–36208.0	
Up-to-7 criteria			0.500
Within	24.7	1.6–23231.0	
Beyond	21.6	1.4–36208.0	
Microvascular invasion			0.252
No	15.5	1.6–23231.0	
Yes	39.5	1.4–36208.0	
Tumor differentiation			0.775
Well	41.8	2.6–7053.0	
Moderate	22.4	1.4–36208.0	
Poor	93.6	2.9–23231.0	
Bilateral tumors			0.698
No	18.8	1.6–36208.0	
Yes	38.0	1.4–17500.0	
Neoadjuvant therapy			0.652
No	21.1	1.4–23231.0	
Yes	31.6	1.6–36208.0	
Salvage liver transplantation			0.293
No	20.6	1.4–23231.0	
Yes	141.0	1.9–36208.0	
Incidental tumors			0.024
No	27.9	1.4–36208.0	
Yes	8.3	3.3–69.2	

**Fig. 1:**
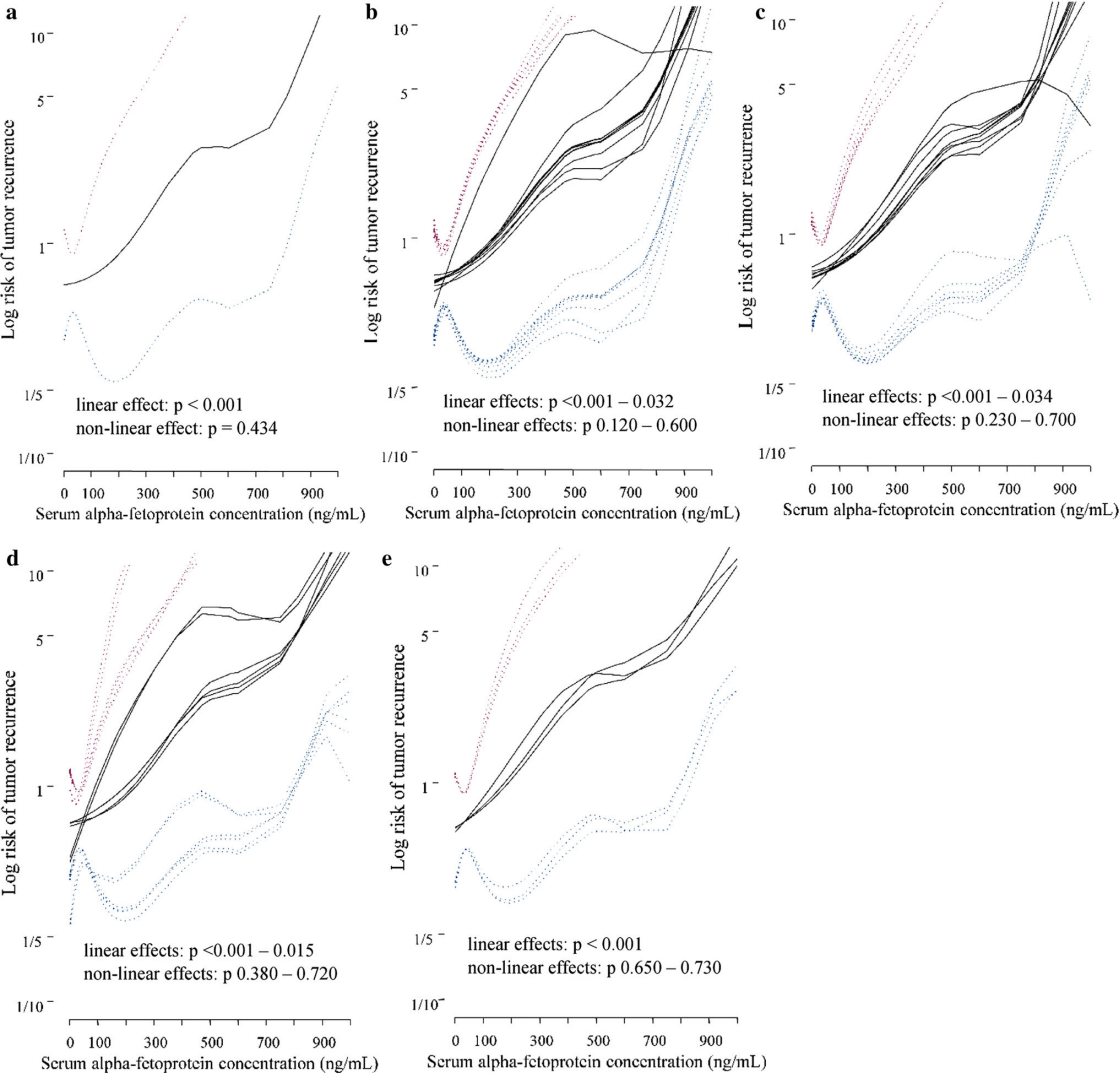
Associations between serum α-fetoprotein (AFP) concentration and the risk of posttransplant tumor recurrence in the univariate analysis (**a**) and adjusted for the impact of potential confounders, including general factors (**b**), tumor-related factors (**c**), intraoperative factors (**d**), and fulfillment of selection criteria (**e**). Risk curves (*solid lines*) are presented with 95 % confidence intervals (*dotted line*s)

AFP was a significant risk factor for tumor recurrence (*p* < 0.001) in the univariate analyses (Table 4). Among the remaining factors, the hazard of recurrence was significantly influenced by the size of the largest tumor (*p* < 0.001), number of tumors (*p* = 0.017), total tumor volume (*p* = 0.001), tumors beyond the Milan criteria (*p* = 0.022) or the Up-to-7 criteria (*p* = 0.021), recipient age (*p* = 0.044), donor sex (*p* = 0.009), and conventional LT technique (*p* = 0.004). Controlled for each of the potential confounders considered in this study, AFP retained significance as a risk factor for posttransplant HCC recurrence in all of the bivariate models (Table 5). Tumors of higher diameter (*p* = 0.010), those beyond the Milan criteria (*p* = 0.044), and female sex of the donor (*p* = 0.029) increased the risk of HCC recurrence independently of the pretransplant AFP level. All associations between AFP and posttransplant recurrence risk were nearly linear in the univariate analyses (linear effect *p* < 0.001; nonlinear effect *p* = 0.434) and bivariate analyses (linear effects: *p* < 0.001–0.034; nonlinear effects *p* *=* 0.120–0.730) (Fig. 1).

**Table 4:** Univariates analyses of risk factors for recurrence during the first three posttransplant years

Factors	HR	95 % CI	*p*
Serum AFP concentration	1.40^a^	1.20–1.63	<0.001
Size of the largest tumor	1.48^b^	1.20–1.83	<0.001
No. of tumors	1.21^c^	1.03–1.41	0.017
Total tumor volume	1.03^d^	1.01–1.05	0.001
Microvascular invasion	1.95	0.63–6.04	0.248
Poor tumor differentiation	2.63	0.54–12.71	0.230
Bilateral tumors	0.70	0.19–2.54	0.586
Neoadjuvant therapy	1.50	0.49–4.62	0.479
Salvage liver transplantation	2.69	0.59–12.20	0.200
Incidental tumors	0.70	0.09–5.40	0.734
Tumors beyond Milan criteria	4.55	1.25–16.67	0.022
Tumors beyond UCSF criteria	2.54	0.85–7.58	0.095
Tumors beyond Up-to-7 criteria	3.64	1.22–10.87	0.021
Recipient age	0.62^e^	0.39–0.99	0.044
Recipient sex	0.76^f^	0.23–2.45	0.641
Child-Turcotte-Pugh class C	1.10	0.21–5.67	0.913
Model for end-stage liver disease	1.03^g^	0.94–1.12	0.561
Hepatitis C virus infection	0.95	0.31–2.91	0.931
Hepatitis B virus infection	1.39	0.47–4.14	0.555
Alcoholic cirrhosis	1.01	0.22–4.56	0.996
Donor age	0.76^e^	0.50–1.16	0.202
Donor sex	0.23^f^	0.08–0.69	0.009
Cold ischemia	1.45^h^	0.84–2.52	0.187
Conventional technique for liver transplantation	4.99	1.68–14.87	0.004
Packed RBC transfusions	0.95^i^	0.78–1.15	0.569
FFP transfusions	0.86^i^	0.66–1.11	0.236
Total operative time	1.16^h^	0.75–1.80	0.503
Duration of hepatectomy	0.63^h^	0.247–1.62	0.340
Warm ischemia	1.05^j^	0.85–1.29	0.662

*HR* hazard ratio, *CI* confidence interval

^a^increase by 100 ng/ml

^b^Increase by 1 cm

^c^Increase by 1 tumor

^d^Increase by 10 cm^3^

^e^Increase by 10 years

^f^Male sex

^g^Increase by 1 point

^h^Increase by 1 h

^i^Increase by 1 unit

^j^Increase by 10 min

**Table 5:** Association between AFP and the risk of tumor recurrence 3 years after exclusion of the impact of potential confounders in a series of bivariate models

Factor 1	HR (95 % CI)^a^	*p*	Factor 2	HR (95 % CI)	*p*
AFP	1.21 (1.02–1.45)	0.032	Size of largest tumor	1.41 (1.09–1.82)^*b*^	0.010
AFP	1.34 (1.13–1.59)	<0.001	No. of tumors	1.10 (0.92–1.31)^*c*^	0.314
AFP	1.46 (1.17–1.82)	<0.001	Total tumor volume	1.02 (0.99–1.04)^d^	0.112
AFP	1.43 (1.20–1.71)	<0.001	Microvascular invasion	1.86 (0.51–6.82)	0.348
AFP	1.43 (1.22–1.69)	<0.001	Poor tumor differentiation	1.92 (0.39–9.44)	0.421
AFP	1.42 (1.21–1.66)	<0.001	Bilateral tumors	0.62 (0.16–2.44)	0.489
AFP	1.40 (1.20–1.63)	<0.001	Neoadjuvant therapy	1.49 (0.44–5.01)	0.524
AFP	1.40 (1.20–1.63)	<0.001	Salvage LT	1.56 (0.19–12.78)	0.681
AFP	1.38 (1.19-1.61)	<0.001	Incidental tumors	—	0.995
AFP	1.31 (1.14–1.52)	<0.001	Milan criteria	8.62 (1.06–71.43)^e^	0.044
AFP	1.36 (1.16–1.58)	<0.001	UCSF criteria	2.34 (0.65–8.47)^e^	0.196
AFP	1.33 (1.14–1.54)	<0.001	Up-to-7 criteria	3.30 (0.89-12.20)^e^	0.074
AFP	1.39 (1.19–1.62)	<0.001	Recipient age	0.82 (0.43-1.57)^f^	0.555
AFP	1.49 (1.26–1.76)	<0.001	Recipient sex (male)	3.10 (0.70-13.81)	0.137
AFP	1.47 (1.24–1.75)	<0.001	Child-Turcotte-Pugh class	1.03 (0.31-3.41)^g^	0.967
AFP	1.39 (1.19–1.62)	<0.001	Model for end-stage liver disease	1.03 (0.91-1.16)^h^	0.647
AFP	1.40 (1.21–1.63)	<0.001	Hepatitis C virus infection	0.84 (0.24–2.90)	0.781
AFP	1.40 (1.20–1.64)	<0.001	Hepatitis B virus infection	1.01 (0.29–3.47)	0.986
AFP	1.40 (1.20–1.63)	<0.001	Alcoholic liver disease	1.35 (0.28–6.44)	0.710
AFP	1.38 (1.16–1.65)	<0.001	Donor age	1.07 (0.66–1.74)^f^	0.791
AFP	1.36 (1.16–1.59)	<0.001	Donor sex (male)	0.24 (0.07–0.86)	0.029
AFP	1.31 (1.08–1.60)	0.008	Cold ischemia	1.43 (0.81–2.54)^i^	0.221
AFP	1.34 (1.14–1.57)	<0.001	Conventional LT technique	2.52 (0.68–9.34)	0.166
AFP	1.33 (1.12–1.58)	0.001	Packed RBC transfusions	0.93 (0.74–1.16)^j^	0.501
AFP	1.37 (1.14–1.64)	<0.001	FFP transfusions	0.84 (0.66–1.06)^j^	0.141
AFP	1.33 (1.10–1.60)	0.003	Total operative time	0.97 (0.63–1.48)^i^	0.883
AFP	1.37 (1.10–1.70)	0.005	Duration of hepatectomy	0.55 (0.21–1.44)^i^	0.226
AFP	1.40 (1.14–1.72)	0.001	Warm ischemia	0.96 (0.79–1.17)^k^	0.696

Each row represents results of separate bivariate analysis

*HR* hazard ratio, *LT* liver transplantation

^a^Increase by 100 ng/ml

^b^Increase by 1 cm

^c^Increase by 1 tumor

^d^Increase by 10 cm^3^

^e^Tumors beyond

^f^Increase by 10 years

^g^Class C

^h^Increase by one point

^i^Increase by 1 h

^j^Increase by 1 unit

^k^Increase by 10 min

**Fig. 2:**
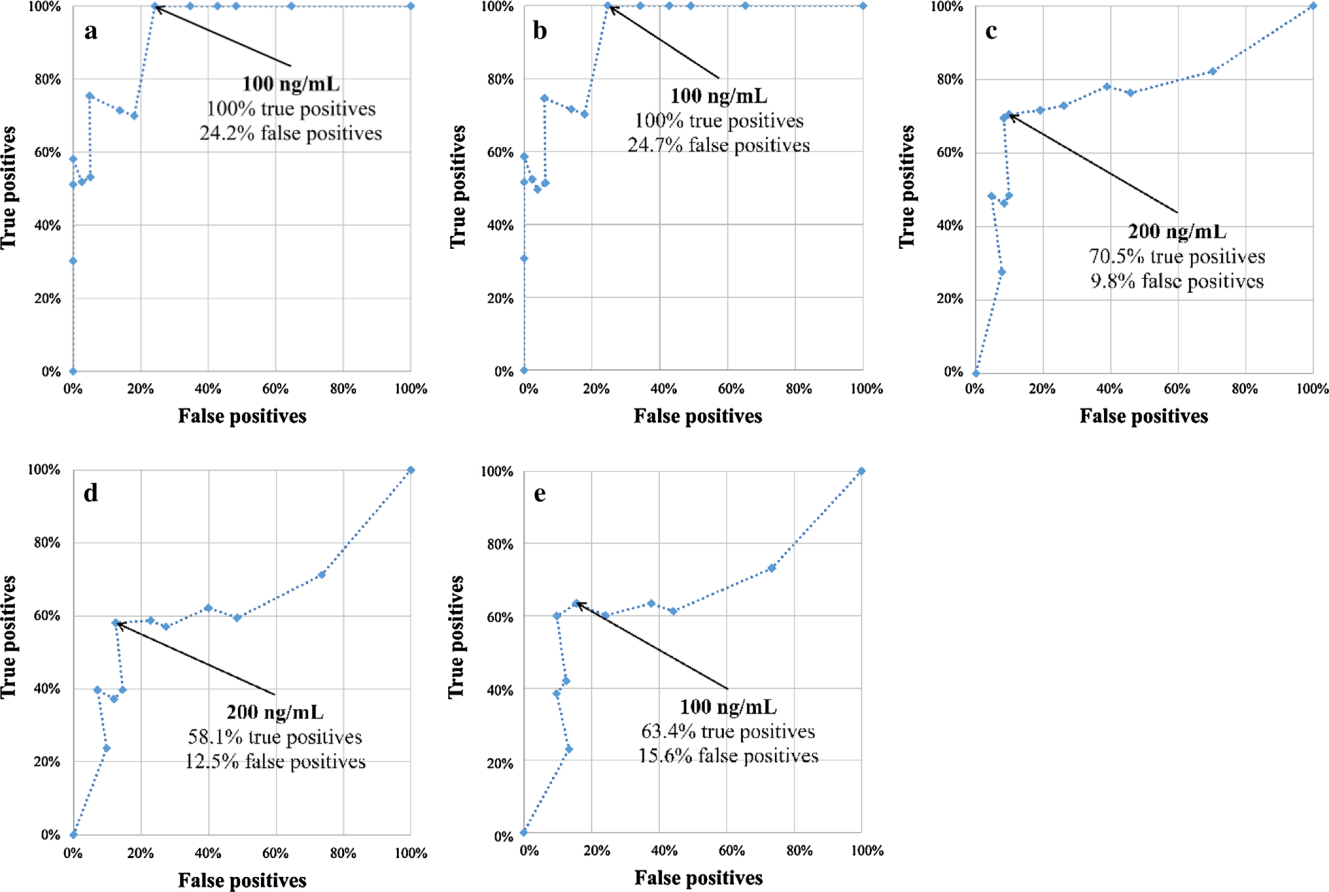
Estimation of the optimal cutoffs for serum AFP concentration for predicting posttransplant tumor recurrence in patients within the University of California, San Francisco (UCSF) (**a**) and Up-to-7 (**b**) criteria and beyond the Milan (**c**), UCSF (**d**), and Up-to-7 (**e)** criteria

More precisely, AFP predicted HCC recurrence in patients beyond (*p* < 0.001) the Milan criteria, within (*p* = 0.001) or beyond (*p* = 0.015) the UCSF criteria, or within (*p* = 0.001) or beyond (*p* = 0.023) the Up-to-7 criteria—but not in patients within (*p* = 0.834) the Milan criteria (Table 6). Thus, AFP cutoffs were estimated for all of these groups except for the latter. Accordingly, the AFP cutoff was 100 ng/ml for patients within the UCSF criteria (TP = 100 %, FP = 24.2 %). The cutoff was the same for patients within (TP = 100 %, FP = 24.7 %) or beyond (TP = 63.4 %, FP = 15.6 %) the Up-to-7 criteria. However, it was 200 ng/ml for patients beyond the Milan criteria (TP = 70.5 %, FP = 9.8 %) and the UCSF criteria (TP = 58.1 %, FP = 12.5 %) (Fig. 2).

**Table 6:** Associations between serum AFP concentration and risk of posttransplant tumor recurrence at 3 years in patients within and beyond the Milan, University of California, San Francisco, and Up-to-7 criteria

Criteria	Factor	HR (95% CI)^a^	*p*
Milan			
Within	AFP	1.09 (0.47–2.53)	0.834
Beyond	AFP	1.32 (1.14–1.53)	<0.001
UCSF			
Within	AFP	1.64 (1.21–2.21)	0.001
Beyond	AFP	1.24 (1.04–1.47)	0.015
Up-to-7			
Within	AFP	1.66 (1.22–2.25)	0.001
Beyond	AFP	1.21 (1.03–1.43)	0.023

^a^Per 100 ng/ml increase

**Fig. 3:**
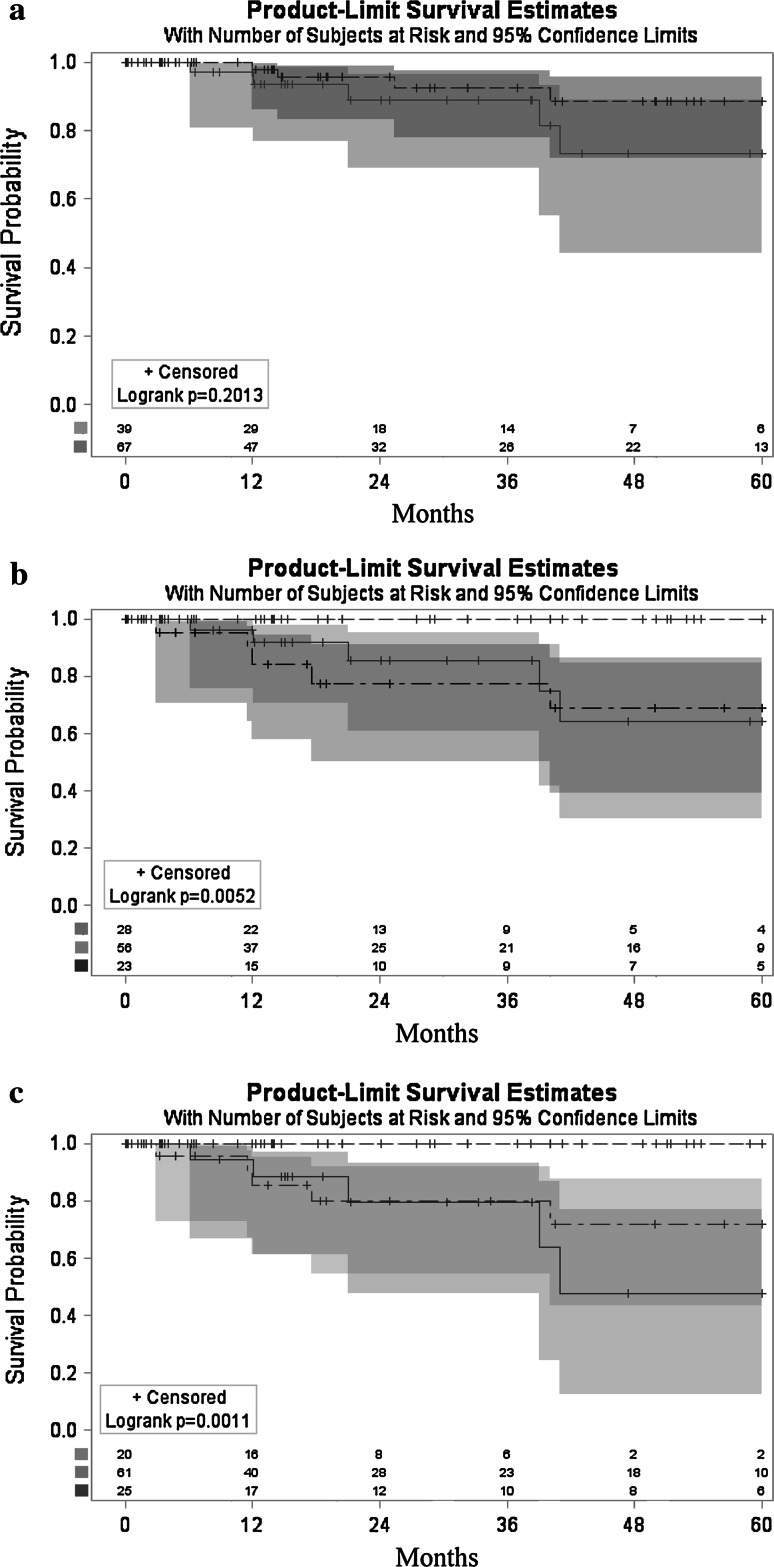
Recurrence-free survivals. **a** Patients within the Milan criteria (*dashed line*) and beyond the Milan criteria (*solid line*) with AFP <200 ng/ml. **b** Patients within UCSF criteria with AFP <100 ng/ml (*dashed line*) and >100 ng/ml (*dashed-dotted line*) and beyond the UCSF criteria with AFP <200 ng/ml (*solid line*). **c** Patients within Up-to-7 criteria with AFP <100 ng/ml (*dashed line*) and >100 ng/ml (*dashed-dotted line*) and beyond Up-to-7 criteria (*solid line*) with AFP <100 ng/ml. Survival curves are presented with 95 % confidence intervals

Recurrence-free survivals at 1, 3, and 5 years after LT were 93.9, 83.6, and 76.8 %, respectively, for the entire study cohort. There was no difference with respect to the 5-year recurrence-free survival between patients within the Milan criteria and those beyond them but with AFP >200 ng/ml (88.8 vs. 75.5 %; *p* = 0.201) (Fig. 3a). Patients within the UCSF criteria and AFP <100 ng/ml exhibited superior recurrence-free survival at 5 years (100 %) compared to patients within these criteria but with AFP >100 ng/ml) (69.0 %) or beyond these criteria and AFP <200 ng/ml (64.3 %; *p* = 0.005) (Fig. 3b). Similarly, the 5-year recurrence-free survival of patients within the Up-to-7 criteria and AFP <100 ng/ml (100 %) was superior to the corresponding rates in patients within and beyond these criteria and AFP >100 ng/ml (71.9 %) or <100 ng/ml (47.8 %), respectively (*p* = 0.001) (Fig. 3c).

## Discussion

The Milan criteria are consistently being criticized for being too restrictive [7, 8, 21, 22]. In fact, it has been shown in several studies that the use of expanded criteria, such as the UCSF or Up-to-7 proposals, might provide results comparable to those provided by the use of Milan criteria [7, 8, 23, 24]. At the same time, large-scale national-level studies indicate that HCC is associated with inferior posttransplant outcomes compared to other indications [9, 10]. In fact, the “Metroticket” concept of the authors of Up-to-7 criteria illustrates that expansion of the limits for morphologic tumor features during selection to undergo LT would further alter the posttransplant outcomes [8]. Moreover, the number of LTs performed for HCC has rapidly increased over the last several years [10]. With the limited pool of deceased donors, this situation might be harmful to patients with other indications for LT because it prolongs their waiting time, resulting in increased deaths while still on the waiting list. Under these circumstances, the HCC Consensus Group stated that the outcomes after LT for HCC and other indications should be comparable [11]. Thus, the optimal selection criteria should ideally bring the risk of posttransplant tumor recurrence to zero.

The results of this study that support the rationale for including AFP in the existing criteria are in line with the findings in previous reports. Notably, both AFP and the size of the largest tumor were independent predictors of tumor recurrence, whereas the number of tumors did not reach the level of significance after adjusting for the impact of the AFP level. However, as the nature of this study was retrospective, such observation clearly does not preclude the importance of the number of tumors during the qualification of patients with HCC for transplantation due to the potential of selection bias. Accordingly, the existing and already popular selection criteria were used to determine the optimal AFP cutoffs subsequently used to develop a strategy for their modification. Analysis of the spline curves revealed linearity of the associations between AFP and the risk of HCC recurrence. Hence, these cutoffs were based on prediction curves derived from Kaplan-Meier recurrence-free survival estimates.

The major finding of the present study is that the estimated rate of tumor recurrence at 5 years was 0 % (corresponding to recurrence-free survival of 100 %) in patients with AFP levels <100 ng/ml and with a tumor burden within the limits of either the UCSF or Up-to-7 criteria. These results support selection of HCC patients with such characteristics for LT either as an addition to the Milan criteria or even their replacement. In both cases, prospective validation of the UCSF or Up-to-7 criteria modified with the 100 ng/ml AFP cutoff in an unselected cohort of HCC patients would be necessary to confirm the rationale for their clinical use. Nevertheless, the proposal for slight expansion of the Milan criteria only to patients with minimal risk of HCC tumor recurrence seems to be a reasonable alternative to expanding the selection criteria to include patients with moderate risk that is comparable to or slightly higher than that provided by the Milan criteria.

Interestingly, recurrence-free survival of patients within the UCSF criteria and AFP levels over the estimated cutoff was similar to those beyond them but with AFP levels <200 ng/ml. In contrast, patients beyond the Up-to-7 criteria and AFP <100 ng/ml exhibited a markedly higher recurrence rate than patients within the Up-to-7 criteria but with AFP >100 ng/ml. Most importantly, tumor recurrences were observed in a remarkable proportion of patients from each of these subgroups. Considering the principle of minimizing the risk of tumor recurrence to achieve outcomes comparable to those seen with other indications, selection of these patients for LT is highly controversial.

Several selection criteria utilizing AFP have been proposed to date, such as the Hangzhou criteria, Seoul criteria, and total tumor volume/AFP (TTV/AFP) criteria. Seoul criteria were defined as the size of the largest tumor ≤5 cm and AFP <400 ng/ml regardless of the number of tumors [25]. The authors of the Hangzhou criteria proposed an identical cutoff. They stated that all patients who had well or moderately differentiated tumors and AFP <400 ng/ml might be considered eligible for LT [26]. However, according to the present study, a cutoff for AFP of 400 ng/ml would lead to a marked increase in the recurrence rate, even in patients who were within the UCSF or Up-to-7 criteria, which are clearly were more restrictive with respect to morphological features than the Seoul or Hangzhou proposals. Conversely, TTV/AFP criteria (TTV < 115 cm^3^ + AFP <400 ng/ml) were associated with superior survival rates in the original study [27] and very low recurrence rate at 5 years in their recent retrospective validation study at the same institution as the authors of the current study [28]. However, as both studies were retrospective and the morphologic limits quite liberal, the results might have been subject to selection bias. Moreover, their fulfillment still did not eliminate the problem of HCC recurrence.

Duvoux et al. [29] also evaluated the concept of combining AFP with morphologic features of the tumor. Based on the AFP levels, tumor size, and number of tumor nodules, the authors of that highly relevant report introduced a predictive model for tumor recurrence that is superior to the Milan criteria for categorizing patients into low- and high-risk groups. In contrast to this interesting proposal of absolute replacement of the Milan criteria with a new risk index, the results of the present study point toward the potential of slight expansion of the former without a negative impact on the risk of recurrence.

In a study based on 92 patients after LT for HCC, Yaprak et al. [30] found that those at low- and high-risk of tumor recurrence can be distinguished based on their AFP level. However, there are several differences between their study and the present study. First, the cutoffs for AFP utilized by Yaprak et al. were arbitrary and hence identical for patients within and beyond the Milan criteria. Notably, the results of the present study indicate that the optimal cutoffs may substantially differ between patients within and beyond particular selection criteria, which seems natural given the correlation between AFP and tumor size. Moreover, the results obtained in the present study highlight the linear nature of the association between AFP and the risk of recurrence, which may prove useful in clinical practice regardless of whether the suggested expansion of selection criteria is adopted. Finally, the results not only confirm the prognostic role of AFP in LT for HCC. They provide additional data to support the rationale for safe utilization of the expanded criteria when limited by the AFP level.

Finally, some authors have suggested that a pretransplant AFP slope is superior to single absolute values [31]. According to a study by Dumitra et al. [32] based on the data of 92 HCC liver transplant recipients, a rising AFP slope was strongly associated with tumor recurrence. However, the positive predictive value of the rising AFP slope was only 25.0 % in that study. On the other hand, results of a large study including 6,817 patients by Merani et al. [33] indicated that the last preoperative AFP level is what matters the most, as patients with stable high AFP and those with originally low but increasing AFP have similar prognoses.

Notably, the usefulness of AFP as an important risk factor for postoperative outcomes was also previously confirmed for patients with HCCs in a noncirrhotic setting. Specifically, Witjes et al. [34] analyzed the data from 94 patients with HCC in noncirrhotic livers and found that AFP and the presence of microvascular invasion were the only independent risk factors for tumor recurrence. Although such patients are not currently considered suitable for LT, these results indicate that AFP should also be taken into consideration when establishing selection criteria for LT in future studies.

Among other potential markers for HCC aggressiveness in a liver transplant setting, des-γ-carboxyprothrombin (DCP) is the one most frequently studied. The results of other studies comparing AFP and DCP as predictors of poor outcomes are contradictory. Hence, there is no clear evidence for superiority of one over another [35, 36]. Unfortunately, pretransplant DCP was not assessed in patients included in the present study.

## Conclusion

Given the minimal risk of posttransplant recurrence, patients who are within Up-to-7 or UCSF criteria should be considered eligible for LT provided the AFP level is <100 ng/ml.
